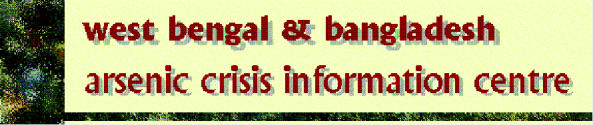# EHPnet: West Bengal & Bangladesh Arsenic Crisis Information Centre

**Published:** 2005-06

**Authors:** Erin E. Dooley

Bangladesh and the neighboring Indian state of West Bengal are the site of what has been called the largest mass poisoning in history: millions of people here are drinking water that is heavily contaminated with arsenic. Researchers, engineers, and others who wish to learn more about this public health crisis can access the latest information and research at the West Bengal & Bangladesh Arsenic Crisis Information Centre, located at **http://bicn.com/acic/**. The page is a service of the Bangladesh International Community News website.

The homepage of the site features a color-coded map showing the levels of arsenic contamination across the region. More than half of Bangladesh’s 10 million drinking water tubewells are contaminated with arsenic in concentrations exceeding World Health Organization guidelines. The United Nations Development Programme estimates that 20,000 people may die of arsenic-related disease each year; however, the numbers are hard to calculate because of the long time it takes for some cancers to emerge. If caught early enough, arsenic poisoning can be reversed with safe drinking water, nutritious foods, and time—three things most people of the region have little of.

The fully searchable site comprises pages of links to news and research articles, data sets, and online forums. Arsenic-crisis and WaterForum are Yahoo group forums that are available to anyone with access to the Internet. Participants may discuss, among other topics, arsenic geochemistry, remediation options, health effects, and related groundwater and surface water issues.

Other pages are devoted to reports, project documents, reference materials, and organizations and individuals from around the world who are involved in the arsenic crisis or related work. For example, a team at Harvard and the Massachusetts Institute of Technology is working to consolidate arsenic data, study the hydro-geochemistry of groundwater, and identify feasible, effective water treatment options for villagers, among other projects.

One page, Water Treatment & Alternative Supplies, specifically links to information on a variety of water treatment projects. Included here are sections on arsenic removal technology verification projects, removal technology providers and projects, alternative water supply technology providers and projects, and field test kits and other measurement-related resources. On another page, Health Effects & Medical Info, visitors will find suggestions for a homemade ointment to ease the suffering of the cracked palms and feet that can accompany chronic arsenic poisoning.

Visitors can also subscribe to *Arsenic Crisis News* through the site. This free newsletter covers such topics as arsenic geochemistry, water treatment technologies, epidemiology, disease mechanisms, and medical treatments. The site provides a list of arsenic-related conferences as well as a bibliography of books and other media, along with ordering information for these resources.

## Figures and Tables

**Figure:**